# Effect of Heating Modes on Reactive Sintering of Ca_3_Co_4_O_9_ Ceramics

**DOI:** 10.3390/ma14020273

**Published:** 2021-01-07

**Authors:** P. Ravi Teja, A. Raja Annamalai, Gecil Evangeline T., Muthe Srikanth, Dinesh K. Agrawal, Chun-Ping Jen

**Affiliations:** 1 Department of Manufacturing Engineering, School of Mechanical Engineering, VIT Vellore, Vellore 632014, India; jagan.raviteja001@gmail.com (P.R.T.); gecilevangeline.t2020@vitstudent.ac.in (G.E.T.); muthe.srikanth@vit.ac.in (M.S.); 2 Centre for Innovative Manufacturing Research, VIT Vellore 632014, India; raja.annamalai@vit.ac.in; 3 Material Research Institute, Pennsylvania State University, Pennsylvania, PA 16802, USA; dxa4@psu.edu; 4 Department of Mechanical Engineering and Advanced Institute of Manufacturing for High-Tech Innovations, National Chung Cheng University, Chia-Yi 62102, Taiwan

**Keywords:** calcium cobaltite, reactive sintering, solid-state synthesis, spark plasma sintering (SPS), electrical properties

## Abstract

The traditional solid-state reaction method was employed to synthesize bulk calcium cobaltite (Ca349/Ca_3_Co_4_O_9_) ceramics via ball milling the precursor mixture. The samples were compacted using conventional sintering (CS) and spark plasma sintering (SPS) at 850, 900, and 950 °C. The X-ray diffraction (XRD) pattern indicates the presence of the Ca349 phase for samples sintered at 850 and 900 °C. In addition, SPS fosters higher densification (81.18%) than conventional sintering (50.76%) at elevated sintering temperatures. The thermo-gravimetric analysis (TGA) and differential thermal analysis (DTA) performed on the precursor mixture reported a weight loss of ~25.23% at a temperature range of 600–820 °C. This current work aims to analyze the electrical properties (Seebeck coefficient (s), electrical resistivity (ρ), and power factor) of sintered samples as a function of temperature (35–500 °C). It demonstrates that the change in sintering temperature (conventional sintering) did not evince any significant change in the Seebeck coefficient (113–142 μV/K). However, it reported a low resistivity of 153–132 μΩ-m and a better power factor (82–146.4 μW/mK^2^) at 900 °C. On the contrary, the SPS sintered samples recorded a higher Seebeck coefficient of 121–181 μV/K at 900 °C. Correspondingly, the samples sintered at 950 °C delineated a low resistivity of 145–158 μΩ-m and a better power factor (97–152 μW/mK^2^).

## 1. Introduction

In current trends, the prime challenge resides in the conversion of dissipated heat to electric energy efficiently. The thermoelectric generators (TEG), also known as the Seebeck generators, are potential players in converting heat flux to electricity through the Seebeck effect. Furthermore, this is analogous in deciding the appropriate prospective thermoelectric (TE) material for power generation from the temperature difference expressed in the dimensionless figure of merit (ZT = S^2^T/ρK). Hence, thermoelectric material possessing a combination of low thermal conductivity (k) and electrical resistivity (ρ) along with a high Seebeck coefficient (S) is hugely preferred. Metal oxides are considered superior TE candidates due to their chemical and thermal stability at elevated temperatures. They also possess unique properties such as high figure of merit (ZT) and long service life at high-temperature ranges [1,2,3]. Therefore, the high-performance TE materials are best suited for a wide variety of applications because of their structural, magnetic, and electronic properties [4].

Calcium cobaltite (Ca349/Ca_3_Co_4_O_9_) is considered a promising thermoelectric oxide material owing to its good thermal and thermoelectric properties, and chemical stability at higher temperatures [5]. Different works reported on calcium cobaltite (Ca349/Ca_3_Co_4_O_9_) by various authors divulge a more in-depth insight into distinct processing inputs and perceived techniques. Qi et al. synthesized Ca349/Ca_3_Co_4_O_9_ that had a stable phase only up to 926 °C and gradually decomposed to Ca326 and cobalt oxide at a reduced temperature [6]. The calcination temperature of the starting mixture mainly depended on calcination time. Traces of Ca326 along with Ca349 was identified at a calcination temperature of 850 °C and more [7,8]. Zhang Feipeng et al. incorporated sol-gel and spark plasma sintering (SPS) to synthesize bulk samples of Ca349 and successfully estimated its TE properties. SPS obtained highly dense samples (~99% of theoretical density) with more improved thermoelectric properties than conventional sintering (CS) [9,10]. Polycrystalline Ca349 powder was synthesized via the sol-gel method, followed by repeated calcination to facilitate calcium carbon decomposition [11]. Different solution methods such as citrate, sol-gel, and polymeric methods were employed to examine the diversity in the preparatory techniques and their effect on porosity, microstructure, and thermoelectric properties. They resulted in a dense and fine-grained ceramic with optimal performance at a high temperature of 800 K [12]. Improvement in the figure of merit (ZT) was closely associated with the influence of nano grain size of Ca349 prepared using sol-gel electrospinning and SPS [13]. K. Agilandeswari et al. synthesized Ca349 through the starch assisted sol-gel combustion method, and Ca349 with a grain size of 150–300 nm were observed at 800 °C with a low electrical resistivity of 1.2 μΩ-cm at 200 °C [14]. The study conducted earlier reports on the influence of grain size and heterogeneity in the porosity of Ca349 on phonon scattering. The nano-sized samples prepared using the sol-gel method evinced a considerable increase in resistivity. As a result, the lower lattice thermal conductivity was high due to grain boundary scattering at interfaces with a better figure of merit (ZT = 0.3) at 1000 K [15].

Sophie Bresch et al. drew the following conclusions by considering various process parameters: (a) The electrical conductivity varied linearly with the rate of densification and (b) the performance of Ca_3_Co_4_O_9_ started to deteriorate at elevated calcination temperature [16]. The characteristics of Ca349 processed using a solid-state reaction (SSR), and SPS at 900 °C for 24 h were not affected by the cooling rate [17]. The performance of calcium cobaltite improved by tailoring the porosity with the addition of K_2_CO_3_ to a classic solid-state sintering technique [18]. Calcium cobaltite is usually prepared by considering CaCO_3_ and Co_3_O_4_ as a precursor mixture and decomposition of carbonates in the air [19]. Polycrystalline Ca_3_Co_4_O_9_ was synthesized using a solid-state reaction and sintering process and reported a monophase of Ca349 at 800 °C and a stable phase up to 900 °C [6,20]. Luxiang Xu et al. employed the SSR method and a cold high-pressure process and then sintered at 1173 K for 48 h under an O_2_ flow with intermediate grindings. It was later pulverized and sintered again at the same temperatures for 12 h [21]. The process parameters had a direct impact on the resultant phase of the cobalites. The incomplete formation of single-phase Ca349 is attributed to the short holding time in reactive sintering [8,22]. Hot pressing (HP) and SPS techniques favored the formation of a dense and textured Ca_3_Co_4_O_9_ ceramic with more enhanced mechanical properties and thermoelectric properties than CS [23]. Thereby, reactive solid-state sintering and SPS techniques are the best option for their lower sintering temperature, shorter holding time, and rapid and uniform heating [24,25,26].

The main objective of this work aims to present the effect of heating modes on the reactive sintering of Ca_3_Co_4_O_9_ ceramics and estimate the electrical properties (Seebeck coefficient (s), electrical resistivity (ρ), and power factor) as a function of temperature.

## 2. Materials and Methods

### 2.1. Solid-State Reaction Method

**Figure 1:**
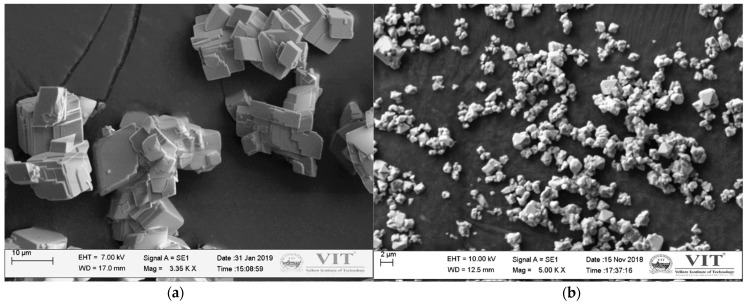
SEM images of the precursors (**a**) calcium carbonate and (**b**) cobalt oxide.

Calcium cobaltite (Ca349) ceramics were processed using the classical solid-state reaction method. The raw materials included calcium carbonate (CaCO_3_) and cobalt oxide (Co_3_O_4_) (SIGMA-ALDRICH, 99% purity, Bangalore, Karnataka, India) of a particle size of less than 10μm as seen in Figure 1. The starting mixture was prepared by amalgamating a stoichiometric amount of CaCO_3_ and Co_3_O_4_ using planetary ball milling for 30 min. Initially, the precursors were ball milled using steel balls (15 No.) in 1:1 g/mL of ethanol taken as a grinding medium. Later the mixture was dried in air for 24 h with subsequent heating at 70 °C for 20 min. The dry powder was consolidated into pellets of 16 mm diameter and 4 mm in height using a hydraulic press at 400 MPa pressure. Furthermore, the green pellets were sintered in a horizontal tubular furnace (OKAY 1400 °C, Model–40T 4Y, Bysakh & Co, Kolkatta, West Bengal, India) at temperatures of 850, 900, and 950 °C at a 5 °C/min heating rate, and 10 h holding time. The starting mixture was synthesized by mixing a stoichiometric amount of CaCO_3_ and Co_3_O_4_, as per the following reaction [7,16]:

(1) $${9\mathrm{CaCO}}_{3}{\;+\;4\mathrm{Co}}_{3}O_{4}{\;+\;O}_{2}\to {3\mathrm{Ca}}_{3}{\mathrm{Co}}_{4}O_{9}{\;+\;9\mathrm{CO}}_{2}$$

### 2.2. Consolidation Using Conventional and Spark Plasma Sintering Techniques

Consequently, the samples were subjected to spark plasma sintering (SPS) at a temperature range of 850–950 °C with an axial pressure (50 MPa) for the heating rate at 100 °C/min, with a 10 min holding time to achieve high density. Thermogravimetric analysis (TGA) and differential thermal analysis (DTA) were carried out on the starting mixture at a heating rate of 20 °C/min for RT-1200 °C using a thermal analyzer (SDT Q600, Lindon, UT, USA). The bulk samples’ phase composition was determined using X-ray diffraction (XRD) (BRUKER D8 Advanced, Yokohama, Japan, Cu Kα, λ = 1.5405 Å). Before XRD analysis, the samples were subjected to polishing using diamond paste in a disc polisher followed by thermal etching at 150 °C below the sintering temperature for 1 h. The conductive layer was then coated with gold–palladium (Au–Pd) on the polished surface using a vapor deposition technique for 1 h. This coating facilitated the electron beam’s penetration during a microstructural examination with a scanning electron microscope (SEM) (Zeiss Penta FET precision, Model: 51-ADD0048, Carl Zeiss Pvt Ltd., Bangalore, India). The samples were diced into 4 × 4 × 12 mm^3^ dimensions for simultaneous measurement of the Seebeck coefficient and electrical resistivity using Ulvac ZEM-3 (ULVAC Technologies, Inc., Methuen, MA, USA) as a temperature function (35–500 °C) in the atmosphere.

## 3. Results

### 3.1. Thermo-Gravimetric Analysis (TGA) and Differential Thermal Analysis (DTA)

**Figure 2:**
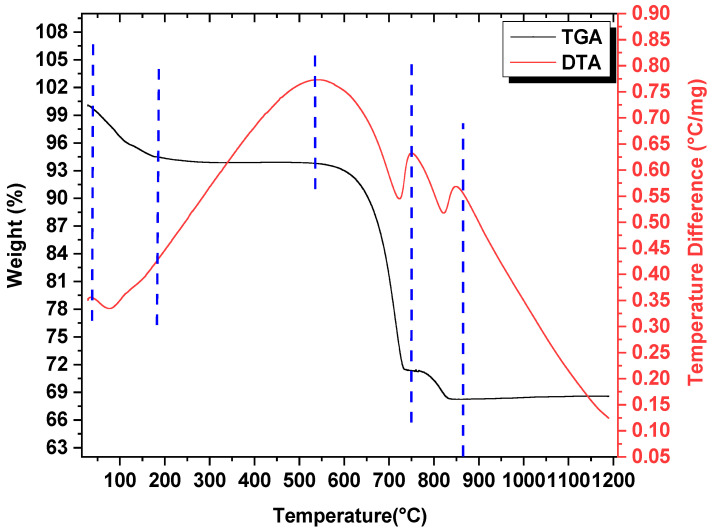
TGA/DTA plots. The DTA curve reaches up to a maximum of 0.78 °C/mg due to the exothermic reaction.

Dynamic TGA/DTA plots are represented in Figure 2. The mixture’s thermal stability was characterized using TGA heated at a rate of 20 °C/min. The precursor mixture weighing 2.46 mg was considered for characterization. A weight reduction of ~5.46% was reported in the first stage due to moisture desorption at a temperature range of 35–180 °C. A significant weight loss of 22.45% + 2.78% in consecutive stages depicted the decomposition of calcium at 600–820 °C, which was as per the following reactions [7,16,27]. The XRD and TGA result confirmed the pure phase of Ca349 reported by earlier works [16].

(2) $${\mathrm{CaCO}}_{3}\to {\mathrm{CaO}\;+\;\mathrm{CO}}_{2}$$

(3) $$2{\mathrm{Co}}_{3}O_{4}\to {6\mathrm{CoO}\;+\;O}_{2}$$

Traces of the Ca349 phase were observed at 600 °C in the TGA graph, indicating the mixture’s elimination of carbon content. Further, the Ca349 phase formation continued up to 820 °C. The single phase of Ca349 was noticed at 800 °C and remained stable up to 926 °C [15,16]. Heating above 926 °C resulted in the decomposition of Ca349 into Ca326 and CoO, as reflected in XRD of the sample sintered at 950 °C. The initial stage of mass loss (0.58%) was confined to the absorbed water content in the precursor mixture (CaCO_3_ + Co_3_O_4_) between 298 and 400 K. The decomposition of calcium carbonate was observed at a temperature between 893–1083 K. The intensive mass loss of ~20.06% in the TGA curve was closely associated with the theoretical mass loss (20.6%) of the equilibrium reaction [12]. The Ca349 phase was observed at 795–812 °C after an endothermic reaction (680–780 °C), as represented in (Figure 2) [28]. The XRD analysis of the sample divulged the phase formation at a calcination temperature of 800 °C.

### 3.2. Relative Density Analysis

The relative densities of the synthesized bulk samples were calculated. The theoretical density of calcium cobaltite (4.567 gm/cc) is listed in Table 1. There was no substantial change except for a ~1% increment in relative density with an increase in sintering temperature (850–950 °C).

Conventional sintering (CS) induced a much lower density for 10 h than SPS, having a relatively high density for 10 min. A high relative density of max 73–74% was achieved after several heating steps at elevated temperatures for hours [18,27]. The reason for the low sintered density (50.76 < 70–74%) was attributed to the Hedvall effect of enhanced chemical reactivity during the phase changes at 900 °C and 950 °C [10,16,23,29]. Higher loss in mass was reported in previous work during reaction sintering (19% for the un-calcined powder to 1% for calcined powder). SPS showed considerable improvement in relative density (79.43% at 850 °C and 81.18% at 950 °C), less than the reported values [10,23,27]. An average of a 31% rise in the relative density of SPS sintered samples processed at the same sintering temperature as that of CS was demonstrated. The simultaneous application of pressure and temperature during the sintering process resulted in the rapid densification of samples.

### 3.3. Material Characterization

#### 3.3.1. Phase Analysis

**Figure 3:**
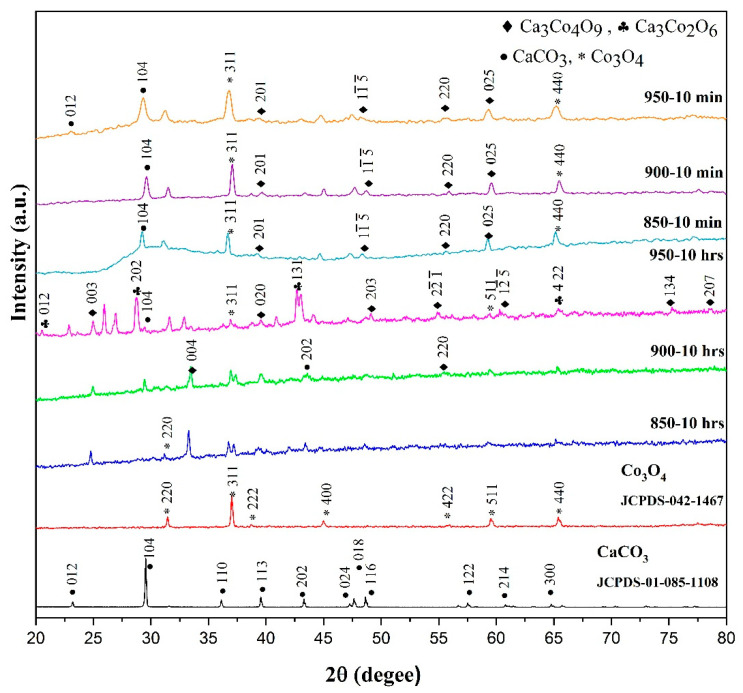
X-ray diffraction (XRD) patterns of bulk samples synthesized at 850, 900, and 950 °C.

The XRD patterns of sintered bulk samples in the 2ϴ range from 20–80° are represented in Figure 3. The obtained peak positions of Ca349 were by the standard JCPDS-023-0110. The diffraction peak at 43.1° corresponded to the precursors, whereas the peaks at 20.5°, 28.47°, and 42.3° were characteristic peaks of Ca326 and identified by standard JCPDS 051-0311. The profile of samples synthesized by SPS matched the standard pattern of Ca349 and minor phases of unreacted precursors (calcium carbonate and cobalt oxide). The formation of single-phase Ca349 depended on a particular holding time and calcination temperature between 800–900 °C [6,15,16,20]. The diffraction peak at 29.5° was characteristic of CaCO_3_. Similarly, peaks at 31.4 °C, 59.5 °C, and 65.5 °C were characteristics of Co_3_O_4_ as per standards JCPDS-85-1108 and JCPDS-65-3103.

#### 3.3.2. Microstructure Analysis

**Figure 4:**
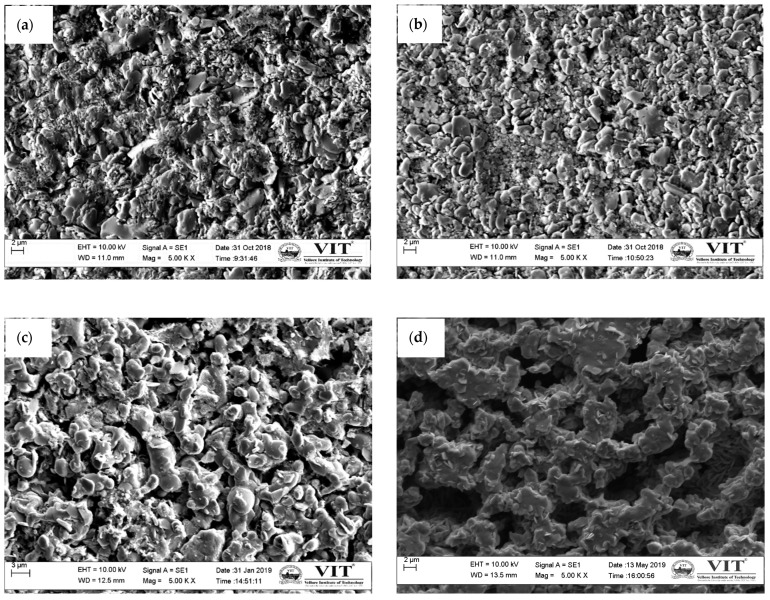

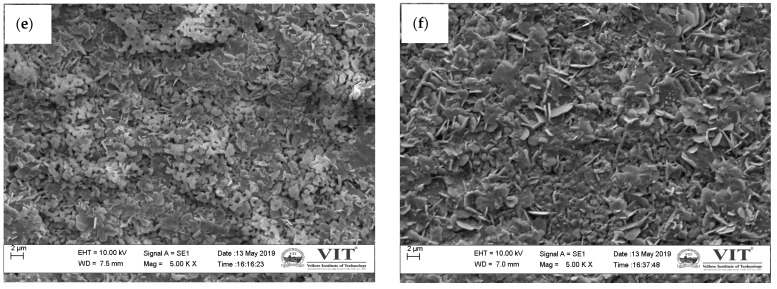
SEM images of bulk samples sintered at (**a**) CS-850, (**b**) CS-900, (**c**) CS-950, (**d**) SPS-850, (**e**) SPS-900, and (**f**) SPS-950.

The SEM images revealed platelet-like grains for conventionally sintered samples at different sintering temperatures, as shown in Figure 4. This type of microstructure was because of the layered crystal structure of Ca349 [16]. The variance in porosity was observed at a temperature range between 850 °C and 950 °C. The oxide ceramics were platelet-shaped but randomly oriented grains with a layered crystal structure. The grain size of the sintered sample was determined by the initial grain size of the precursor powder. The samples sintered at 850 °C reported a porosity that gradually reduced with an increase in temperature at 900 °C. The elemental analysis (EDAX) was as per the XRD profile of calcium carbonate. The carbon content in samples sintered at 850, 900, and 950 °C by both CS and SPS had 1.44 wt.%, 2.31 wt.%, and 1.76 wt.% respectively. The existence of unreacted precursors (calcium carbonate and cobalt oxide) estimated by XRD analysis was validated using the elemental analysis performed on samples sintered by SPS at 850, 900, and 950 °C. The carbon content (1.80, 5.28, and 3.38 wt.%) of samples sintered by SPS at 850, 900, and 950 °C are listed in Table 2. SPS samples possessed tiny grain structures that were mostly oriented perpendicular to the applied pressure. Meanwhile, irregular shapes with plate-like grains and sharp edges with random orientation were observed for samples sintered at 950 °C.

### 3.4. Electrical Properties

#### 3.4.1. Seebeck Coefficient

**Figure 5:**
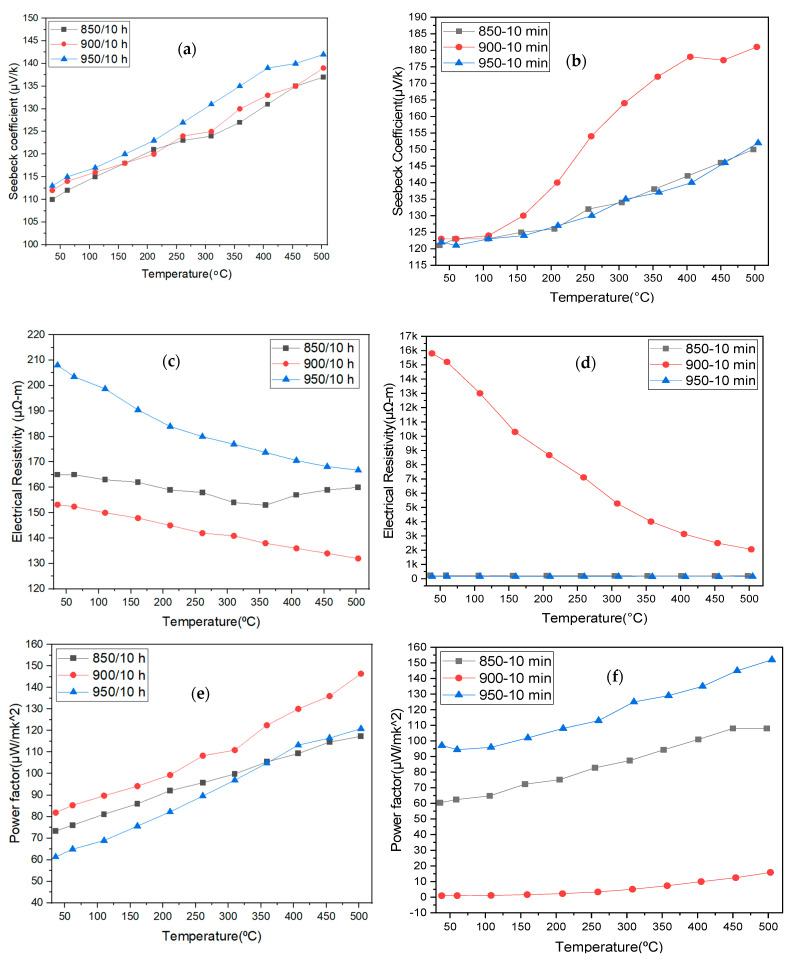
Electrical properties of sintered bulk samples (**a**,**c**,**e**) are conventional sintered, and (**b**,**d**,**f**) are SPS sintered samples.

The synthesized bulk samples exhibited a positive Seebeck coefficient at different sintering temperatures because of the dominant hole inferred from Figure 5. In Figure 5a for CS, a slight difference in the Seebeck coefficient with a rise in sintering temperature was noted. A higher Seebeck coefficient of 113–142 μV/K, relatively higher than reported values at 950 °C, was recorded [10,13,29,30]. The Seebeck coefficients were non-sensitive to sintering techniques, temperature, and density [16]. At room temperature, the SPS samples exhibited a minimum value of 121 μV/K, shown in Figure 5b. The specimens sintered at different temperatures reported a result closer to the typical value (123 μV/K) at 900 °C for 24 h [2,24]. The sample sintered at 900 °C attained a maximum of 181 μV/K at 500 °C. Significantly, the other two samples exhibited the highest Seebeck coefficient of ~152 μV/K at the maximum testing temperature. A classical sintered Ca349 at 920 °C displayed a Seebeck coefficient of 97–135 μV/K, and 114–142 μV/K by hot pressing (800 °C for 16 h) [25]. Samples processed by CS, SPS, and HP through the solid-state sintering method of the same composition mixture reported approximately the same Seebeck coefficient with the highest value of 170 μV/K at 527 °C [22].

#### 3.4.2. Electrical Resistivity

The electrical resistivity was reduced to (132 μΩ-m) with an increased working temperature at 500 °C [13]. In the SPS process, the samples sintered at 950 °C exhibited minimum electrical resistivity between 145–158 μΩ-m lower than the CS process at 900 °C. No remarkable changes (±4 μΩ-m) were observed in the rise in temperature from room temperature to 500 °C. The sample sintered classically at 920 °C exhibited a relatively constant electrical conductivity of 33 S/cm (~303 μΩ-m) throughout a temperature range from room temperature (RT) to 500 °C [25]. In the case of Ca349 synthesized by CS, the positive Seebeck coefficient was due to the dominant hole concentration.

#### 3.4.3. Power Factor

The thermoelectric material’s performance for different sintering methods and the temperature were estimated using the numerical expression mentioned below. It was observed that material with a high power factor retained high thermoelectric performance.

Power factor (α) = S^2^/ρ (μW/mK^2^) (4)

where

S—Seebeck coefficient (μV/K); and

Ρ—Electrical resistivity (μΩ-m)

The power factors of ~81.9 and 146 μW/mK^2^ were achieved at room temperature and 500 °C [30]. An appreciable increase in power factor was observed at 850 °C and 900 °C, corresponding to the phase formation of Ca349 [14]. Ca326 at 950 °C was responsible for a low range value of 61–120 μW/mK^2^. The samples prepared using SPS at 950 °C exhibited a power factor of 97–152 μW/mK^2^ better than those calcined twice and sintered at 900 °C for 24 h using CS [25]. SPS reported better results when compared to CS, even after multiple calcinations. As per numerical expression, it was estimated that ceramics generated a high power factor for low electrical resistivity. The samples sintered at 850 °C and 950 °C yielded a better power factor in our work since the power factor of Ca349 directly impacted the electrical resistivity than the Seebeck coefficient.

## 4. Discussion

In this research work, the samples sintered using spark plasma sintering (SPS) manifested significant densification (roughly 30%) at a shorter duration than conventional sintering. The TGA/DTA studies on the precursor mixture divulge a weight loss of 25.23% that was confined to the precursors’ reaction at 600–820 °C, which resulted in the formation of the Ca349 phase. The XRD pattern and SEM observation more evidently indicated the presence of the Ca349 phase predominantly at 850 °C and 900 °C. On the other hand, the sample sintered at 950 °C unveiled the existence of Ca326 in the XRD pattern due to the decomposition of Ca349 at 950 °C. The enhanced Seebeck coefficient of the SPS samples (97–152 μW/mK^2^) was due to the high densification of Ca349. These samples reported a better power factor by yielding a low electrical resistivity.

## Figures and Tables

**Table 1:** Comparison of relative density of CS and SPS Ca_3_Co_4_O_9_ samples at 850, 900, and 950 °C.

Sintering Temperature (°C)	CS	SPS
850	47.81	79.43
900	49.71	80.52
950	50.76	81.18

**Table 2:** Elemental analysis of Ca_3_Co_4_O_9_ using EDAX.

Elements	Conventional Sintering Weight %			Spark Plasma Sintering Weight %		
	850 °C—10 h	900 °C—10 h	950 °C—10 h	850 °C—10 min	900 °C—10 min	950 °C—10 min
C (K)	1.44	2.31	1.76	1.80	5.28	3.38
Ca (K)	21.37	21.34	25.74	22.80	27.95	23.57
Co (L)	45.45	47.37	38.71	46.23	31.37	40.93
O (K)	31.74	28.98	33.79	29.17	35.40	32.12

## Data Availability

The data that support the findings of this study are available from the corresponding author upon reasonable request.
